# Dacryocystorhinostomy videos on YouTube as a source of patient education

**DOI:** 10.1007/s10792-024-03139-0

**Published:** 2024-04-23

**Authors:** Gurfarmaan Singh, Raghav Goel, Yinon Shapira, Joseph Hewitt, Christopher Ovenden, Dinesh Selva

**Affiliations:** 1 The University of Adelaide, North Terrace, Adelaide, SA 5000 Australia; 2 Department of Ophthalmology, Royal Adelaide Hospital, Port Road, Adelaide, SA 5000 Australia

**Keywords:** Ophthalmology, Dacryocystorhinostomy, YouTube, Health education, Videos

## Abstract

**Background:**

To determine the quality and reliability of DCR YouTube videos as patient education resources and identify any associated factors predictive of video quality.

**Methods:**

A YouTube search was conducted using the terms “Dacryocystorhinostomy, DCR, surgery” on 12th of January 2022, with the first 50 relevant videos selected for inclusion. For each video, the following was collected: video hyperlink, title, total views, months since the video was posted, video length, total likes/dislikes, authorship (i.e. surgeon, patient experience or media companies) and number of comments. The videos were graded independently by a resident, a registrar and an oculoplastic surgeon using three validated scoring systems: the Journal of the American Medical Association (JAMA), DISCERN, and Health on the Net (HON).

**Results:**

The average number of video views was 22,992, with the mean length being 488.12 s and an average of 18 comments per video. The consensus JAMA, DISCERN and HON scores were 2.1 ± 0.6, 29.1 ± 8.8 and 2.7 ± 1.0, respectively. This indicated that the included videos were of a low quality, however, only DISCERN scores had good interobserver similarity. Videos posted by surgeons were superior to non-surgeons when considering mean JAMA and HON scores. No other factors were associated with the quality of educational content.

**Conclusion:**

The quality and reliability of DCR related content for patient education is relatively low. Based on this study’s findings, patients should be encouraged to view videos created by surgeons or specialists in preference to other sources on YouTube.

## Introduction

Dacryocystorhinostomy (DCR) is a common surgical procedure to relieve symptoms of epiphora (watery eyes) secondary to nasolacrimal duct obstruction (NLDO) [1]. The incidence of NLDO has been reported to be 20.24 per 100,000 [2].

Medical information relating to surgical procedures is often complicated which can cause patients to feel overwhelmed after being provided information by their doctor [3]. A systematic review of literature in relation to patients understanding of their surgeries, found that only 29% (6/21) of studies noted an acceptable understanding of procedure related information by participants [4]. The lack of understanding in the case of DCR is likely due to the complexity of steps involved in the procedure and the intricate anatomy. This may result in patients seeking additional information from multimedia platforms such as YouTube.

Online information searches are a common source of medical information for patients, with YouTube being a popular resource [5–7]. At present, there is no regulation or peer review process required to be undertaken prior to uploading of health-related videos on the platform. Therefore, if the reliability and quality of uploaded videos are of a low standard, there exists a risk of patients understanding incorrect or biased information. The educational quality of YouTube videos for patients has previously been explored, with most studies reporting content to be of low quality across a diverse range of medical and surgical procedures [8–11].

To our knowledge this is the first study assessing the educational quality of DCR related videos on YouTube. We aim to determine the quality and reliability of DCR surgery videos using validated scoring systems. In addition, we aim to determine if any factors are predictive of higher quality educational content for patients.

## Methods

A YouTube search was conducted using the words “Dacryocystorhinostomy, DCR, surgery” on 12th of January 2022. The search was conducted in English (United States), with no filters applied to the search criteria. The first 50 relevant videos that were in English language, at least 6 months old and not duplicated were selected for inclusion in this study.

For each video the following was collected: a video hyperlink, title, total views, months since the video was posted, video length in seconds, total likes, total dislikes, authorship (i.e. surgeon, patient experience or media companies) and number of comments.

The videos were graded independently by an ophthalmology resident, registrar and an oculoplastics specialist surgeon. Each assessor scored the 50 videos using three ranking systems: the Journal of the American Medical Association (JAMA), DISCERN, and Health on the Net (HON). A sample size of 50 videos was used to maintain consistency with previously published literature assessing the quality of YouTube videos as a source of patient education [8, 12–15]. The JAMA criteria is scored on the basis of four categories (i.e., authorship, attribution, currency, and disclosure) and assigned a score out of four [16]. DISCERN is a detailed questionnaire consisting of 16 questions, with a score of 5 being assigned for each section and a mean being calculated in order to arrive at a final score [17]. The HON system consists of eight criteria such as authoritativeness, complementarity, privacy and advertising policy [18]. In this system each category is scored either 0 or 1.

In this study the consensus JAMA, DISCERN and HON were calculated as means ± SD. In order to determine interobserver reliability, interclass correlation of scoring (ICC) was undertaken, with a score of < 0.40 being considered poor, 0.40–0.59 fair, 0.60–0.74 good and > 0.75 excellent. The mean JAMA, HON and DISCERN scores were then used as the consensus scores for all further analyses. Linear regression was employed to determine if there was an association between scores to: view count, months online, video length, positivity (likes/likes + dislikes) and number of comments. Association between authorship (i.e., surgeon versus non-surgeon) and JAMA, DISCERN and HON scores was assessed using independent sample t-tests. In all statistical analysis *P* < 0.05 were considered statistically significant.

## Results

**Fig. 1:**
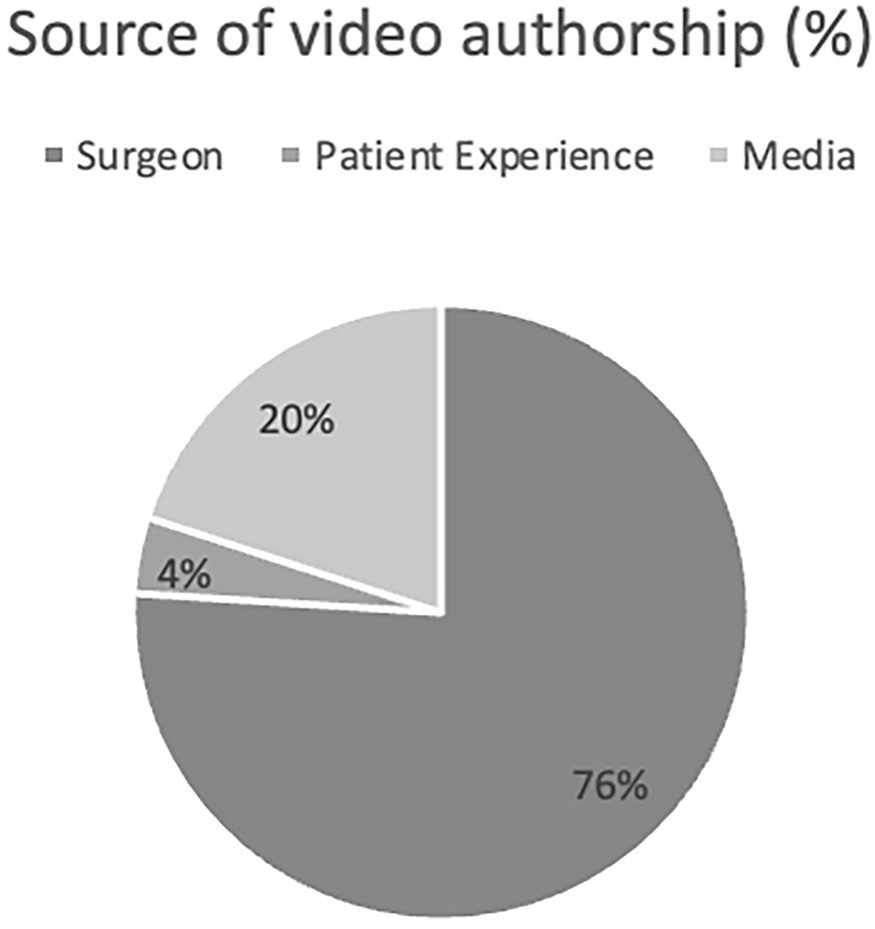
Source of video authorship for included videos

The total number of views was 708,406 across the 50 selected videos, with a mean of 22,992 ± 37,396 views per video (range 305–198,477). The mean number of months the videos had been published online was 64.87 ± 27.22 (range 20.65–137.52). The average length of the included videos was 488.12 ± 472 s (range 15–2035), and all videos had a mean of 18 ± 76 comments (range 0–539). The overall positivity (i.e., likes on the video compared to dislikes) of all selected videos was approximately 95.3%, with 76% of videos being created by surgeons or ophthalmologists (see Fig. 1).

The consensus JAMA, DISCERN and HON scores were 2.1 ± 0.6, 29.1 ± 8.8 and 2.7 ± 1.0, respectively. In this study, only the DISCERN scoring system had an ICC value > 0.75 (0.852). The JAMA and HON systems correlations produced scores of 0.544 and 0.578, respectively.

The highest mean score for the included videos when considering the JAMA criteria was “currency” and the lowest was “attribution” (see Table 1 for mean scores of each JAMA criteria). For the HON criteria, “confidentiality” had the highest mean score and “justifiability” has the lowest mean score (See Table 2). From the 16 DISCERN questions; question 2- “does the video achieve its aims,” was found to have the highest mean score for included videos and question 12- “Does it describe what would happen if no treatment is used” had the lowest mean score (Table 3).

**Table 1:** Mean scores of individual JAMA criteria

Criteria^a^	Authorship	Attribution	Currency	Disclosure	Total mean score
Mean	0.81	0.09	0.99	0.20	2.10
SD (±)	0.39	0.29	0.08	0.40	0.60

^a^In this system each category is scored either 0 or 1

**Table 2:** Mean scores of individual HON criteria

Criteria^a^	Authoritativeness	Complimentary	Confidentiality	Attribution	Justifiability	Transparency	Financial disclosure	Advertising	Total mean score
Mean	0.71	0.27	0.83	0.23	0.10	0.27	0.15	0.12	2.70
SD (±)	0.46	0.44	0.37	0.42	0.30	0.44	0.35	0.33	1.00

^a^In this system each category is scored either 0 or 1

**Table 3:** Mean scores of individual DISCERN criteria

Criteria^a^	Are the aims clear?	Does it achieve its aims?	Is it relevant?	Is it clear what sources of information were used to compile the publication (other than the author or producer)?	Is it clear when the information used or reported in the publication was produced?	Is it balanced and unbiased?	Does it provide details of additional sources of support and information?	Does it refer to areas ofuncertainty?	Does it describe how each treatment works?	Does it describe the benefits of each treatment?	Does it describe the risks of each treatment?	Does it describe what would happen if no treatment is used?	Does it describe how the treatment choices affect overall quality of life?	Is it clear that there may be more than one possible treatment choice?	Does it provide support for shared decision-making?	: Based on the answers to all of the above questions, rate the overall quality of the publication as a source of information about treatment choices	Total Mean Score
Mean	2.67	2.9	2.3	1.41	2.35	1.86	1.45	1.31	2.39	1.73	1.4	1.26	1.35	1.52	1.4	1.83	29.1
SD (±)	1.03	1.07	1.01	0.80	1.21	0.75	0.89	0.56	0.85	0.99	0.89	0.70	0.78	1.03	0.93	0.84	8.80

^a^In this system each category is scored between 1 and 5

**Fig. 2:**
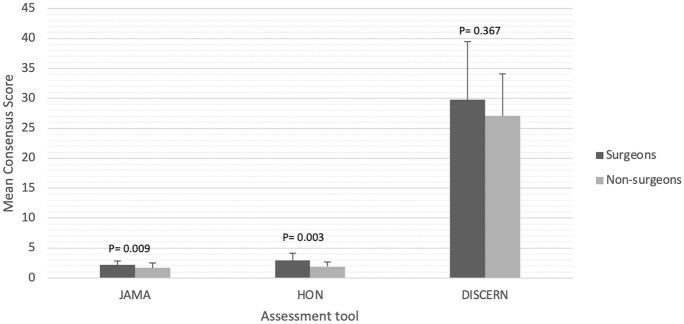
Mean assessment tool consensus scores for surgeons versus non-surgeons

The following variables demonstrated no association with JAMA, DISCERN or HON scores: view count, months online, video length, positivity, and number of comments. The only variable demonstrating an association was video authorship (surgeons versus non-surgeons). Videos uploaded by surgeons were noted to have a higher JAMA and HON scores, with *p*-values < 0.05 (see Fig. 2). Interestingly, DISCERN scores demonstrated no significant difference when posted by surgeons versus non-surgeons (*P* = 0.367).

## Discussion

In this study, the average quality of DCR surgery educational videos, using three validated assessment tools, was poor. We found 76% of videos were authored by surgeons, and two assessment tools (JAMA and HON) demonstrated these videos were superior to those posted by non-surgeons.

In ophthalmology, several studies have assessed the quality of YouTube educational resources, concluding that the overall quality is low [12, 19–22]. In cataract surgery, Bae et al.’s [12] findings were similar to our study, with 71% of videos produced by medical professionals, and the overall educational quality of videos was deemed low. Mangan et al. [21] focussed on strabismus educational content and found that only 28.5% of videos were produced by ophthalmologists, with videos published by academic institutions having the highest quality and reliability scores. In the context of oculoplastics, the quality of eyelid blepharoplasty videos has been assessed. Karataş et al. [22] reported 79% of videos were uploaded by physicians, with higher DISCERN scores compared to our study (45.06 ± 12.88) and lower JAMA scores (1.39 ± 1.06). Garip et al. [23] evaluated eyelid ptosis surgery, they also concluded the available videos were not a reliable source of educational information. Similar to our study, videos created by physicians or surgeons were of higher quality and educational value.

Noteworthy, the benefit of certain videos is not considered within the scope of the assessment tools employed. Although some videos may score well on the basis of the scoring criterion, these tools do not assess the specific content within the videos. Therefore, whilst a study may contain all technical aspects (e.g. date, author), it may lack the correct information (e.g. surgical steps involved). The tools also do not consider the relevance to the specific user, i.e. medical professional versus patient. Furthermore, the tools do not account for the emotional impact a video may have on a patient, for example if a reported experience is negative, it may disproportionality increase a patient’s concerns, anxiety or perceived risk of complications related to a surgical procedure. Another consideration is that none of the assessment tools used consider if all the information related to a procedure is present within the specific video. Given this, there may be a need to develop a dedicated assessment tool for surgical education videos which incorporates all the factors above. Additionally, the DISCERN and JAMA scoring systems are specifically designed for assessment of the quality of written materials. Therefore, the validity of these tools when applied to multimedia educational material (i.e. YouTube videos) is not known.

Winker et al. [24] have previously published guidelines for authors on uploading medical and health related information on the internet. Many of the assessment tools’ criteria are included in these guidelines including the date content was posted and the source of funding or ownership. When considering the quality of content, the guidelines recommend some form of external peer review process by experts in the field, this is a particular aspect lacking in the assessment tools employed. In future, these guidelines may be used as the basis for the development of an assessment tool to precisely evaluate the quality of surgical education videos. An adaption of the Ensuring Quality Information for Patients (EQIP) tool used for assessing the quality of written medical information (e.g. pamphlets) could be employed for online educational resources [25]. This would have to be with the caveat of the tool being validated prior to its widespread implementation.

A limitation of our study is that it only assessed the first 50 videos related to DCR surgery. Therefore, our findings regarding the quality and reliability of DCR related videos as patient education content may not necessarily be able to be extrapolated to videos beyond the first 50. It is, however, most likely for a patient to select one of the first 50 videos to view as a source of further information on their DCR procedure. Furthermore, YouTube’s algorithm for displaying the first 50 videos on each search of DCR surgery is not entirely predictable or consistent. As a result, some videos assessed in this study may not appear in a randomly selected patient’s top 50 searches. Moreover, only videos in English were reviewed; hence it is impossible to conclude the quality of educational videos in other languages. This limits the utility of our findings in non-English speaking regions, as searches in these geographic locations may yield a significantly different set of videos.

In this study only the DISCERN ICC was considered excellent, suggesting scores by all three assessors were very comparable. The JAMA and HON scores ICC was considered fair. This suggests there was more inter-assessor variability when scoring according to these systems within this study, thereby limiting their reliability in the context of this study. Previous studies have also demonstrated lower ICC scores for HON and JAMA [8, 9]. A possible explanation may be these criteria have only 2 options (yes or no), with less questions than DISCERN. This may result in the impact of assessors disagreeing becoming more significant. Furthermore, the DISCERN tool provides more context and explanation for each criterion, limiting the impact individual interpretation of questions by assessors on the overall score.

## Conclusion

YouTube provides patients with easy access to a range of DCR surgery related videos. The quality and reliability of DCR related content for patient education is relatively low. Given the current trend for patients to seek additional information on the internet, specialists should consider counselling patients on the limited quality of DCR surgery content on YouTube and provide factual information through pamphlet or college instituted videos. Furthermore, based on this study’s findings, patients should be encouraged to view videos created by specialists in preference to other sources of content. Finally, there is a strong need to devise a consensus on a dedicated scoring tool for online educational videos. This assessment should include assessing the appropriateness, accuracy and completeness of the provided information pertaining to the specific treatment being discussed.
